# Interaction between body mass index and family history of cancer on the risk of female breast cancer

**DOI:** 10.1038/s41598-024-54762-x

**Published:** 2024-02-28

**Authors:** Jiamin Cao, Jun Li, Zuofeng Zhang, Guoyou Qin, Yi Pang, Mengyin Wu, Kai Gu, Huilin Xu

**Affiliations:** 1 Shanghai Minhang Center for Disease Control and Prevention, No. 965, Zhongyi Road, Shanghai, 201101 China; 2 Shanghai Municipal Center for Disease Control and Prevention, No. 1380, Zhongshan West Road, Shanghai, 200336 China; 3 Department of Epidemiology, Fielding School of Public Health, University of California, Los Angeles, CA USA; 4 School of Public Health, Fudan University, Shanghai, China

**Keywords:** Body mass index, Breast cancer incidence, Family history of cancer, Interaction, Diseases, Risk factors

## Abstract

Both body mass index** (**BMI) and family history of cancer are established risk factors for female breast cancer. However, few studies explored the potential interaction between both factors. We assessed the association of BMI and its interaction with family cancer history on the risk of female breast cancer in Shanghai, China. Based on a population-based prospective cohort study started from 2008 to 2012 with 15,055 Chinese female participants in Minhang district, Shanghai. Cox regression models were used to estimate the association of BMI and its interaction with a family history of cancer on breast cancer risk. The additive interaction was evaluated by the relative excess risk due to interaction (RERI) and the attributable proportion due to interaction (AP), and the multiplicative interaction was assessed by the product term (BMI* family history of cancer) in the Cox regression model. Compared with BMI of < 24 kg/m^2^ and no family history of cancer, women with BMI of ≥ 24 kg/m^2^ and a family history of cancer had a higher risk for breast cancer with HR 2.06 (95% CI 1.39, 3.06). There was an additive interaction between BMI and family history of cancer on breast cancer incidence, with the RERI being 0.29 (95% CI 0.08, 0.51) and the AP being 0.37 (95% CI 0.08, 0.66). The coexistence of obesity and cancer family history may exacerbate breast cancer incidence risk, highlighting the importance of weight management in women with a family history of cancer.

## Introduction

Breast cancer is the most commonly diagnosed cancer worldwide, with cases expected to reach 4.4 million by 2070^1,2^ Among women, breast cancer accounts for approximately 25.8% of all cancer cases and 15.6% of cancer deaths based on GloboCan 2020^1,3^. Recent socioeconomic developments and lifestyle changes in some Asian countries, including China, have led to an increase in breast cancer incidence rate^4^. Breast cancer is now the most common cancer among Chinese women, with cases in China accounting for 12.2% of newly diagnosed cases worldwide^5–7^. As a result of the high incidence of breast cancer, not only women will be burdened with this disease, but also families and society will bear the high expense of medical treatment^8^.

Among women, obesity and a family history of cancer are both established risk factors for breast cancer^9–11^. Women with a high body mass index (BMI) have a much higher risk of breast cancer than those with a normal BMI^12^. A study showed that with each 5 kg/m^2^ increase in BMI, the risk of breast cancer increased by 31%^13^. The effect of family history of cancer as an unmodifiable risk factor for breast cancer has also been explored by many studies, with the population-attributable risk proportion of family history of cancer being 8.7%^14,15^. Although the relationship between obesity and family cancer history on breast cancer has been well established, the interaction between BMI and family cancer history on breast cancer remains unclear. A good understanding of the interaction is vital to identify target groups for weight management advice and effectively reduce the risk of breast cancer.

In this prospective population-based cohort study, we investigated the possible nonlinear association between BMI and the incidence of breast cancer in women. On this basis, we further explored the interaction of BMI and cancer family history on the incidence of breast cancer and quantified the increased risk of breast cancer caused by a higher BMI in women with a family history of cancer. The study is essential to clarify whether there is an additional benefit of interventions for obesity among female individuals with a family history of cancer.

## Materials and methods

### Study population

This population-based prospective cohort study was based on a community colorectal cancer screening program implemented in an urban–rural integration town named Qibao, located in Minhang District, south-eastern Shanghai, China^16^. 15,101 female participants were initially enrolled in the study from 2008 to 2012. We excluded 46 participants with follow-up durations of less than 3 months (N = 9), missing demographic information (N = 30), and missing information on exposures and other covariates (N = 7). Ultimately, 15,055 participants were included in this study. All participants were followed up until the date of cancer diagnosis, death, or loss to follow-up, or June 30, 2021.

### Data collection

During the baseline recruitment period, data were collected through face-to-face interviews by trained interviewers using a standard questionnaire that included demographic and lifestyle factors such as sex (male, female), birthday, education level (primary, secondary, tertiary), marital status (married/remarried, divorced/separated/widowed/unmarried), 3-month income level (< 2000¥, 2000–4000¥, ≥ 4000¥), smoking status (yes, no), drinking status (yes, no), frequency of eating preserved/fried and smoked/high-fat foods per week (at least once, not even once) and intake of fruits and vegetables per day (< 300 g, ≥ 300 g). Data on the diagnosis of type 2 diabetes (yes, no) were identified through linkage to the Diabetes Standardized Management Program (DSMP) based on the local electronic health record management system (EHR)^17^.

According to the standard protocol, height and weight were measured without shoes and light clothing, and BMI was calculated as weight (kg) divided by the square of height(m). The BMI cut-off for Chinese individuals proposed by the China Obesity Task Force was used to classify participants into four groups: underweight, < 18.5 kg/m^2^; normal weight, 18.5–23.9 kg/m^2^; overweight, 24–27.9 kg/m^2^; and obesity, ≥ 28 kg/m^2^^18^. Family history of cancer was divided into family history of cancer (including parents, siblings, children, second-degree relatives, and collateral relatives with cancer) and no family history of cancer according to baseline self-report data.

### Definition of outcome

The outcome of interest in this study was the incidence of breast cancer cases (Classification of Diseases and Related Problems, 10th Revision, Clinical Modification, C50) during the study period. Until June 30, 2021, newly diagnosed breast cancer patients were identified using a record linkage system with the Shanghai Cancer Registry and Shanghai Vital Statistics through the Chinese Resident Identity Card number^19^. Person-years of follow-up were calculated from the date that participants were first investigated to the date on which breast cancer was diagnosed, or death date, or June 30, 2021, whichever occurred first.

### Statistical analyses

Participants were grouped by the category of baseline BMI, and baseline characteristics were presented as the median (interquartile range) for continuous variables and as the frequency (%) for categorical variables. The χ^2^ test for categorical variables and the Kruskal‒Wallis test for continuous variables were used to compare demographic characteristics and lifestyle factors. We presented the cumulative incidence risk of breast cancer across baseline BMI classifications.

We presented the crude incidence rate(CIR) of breast cancer across baseline BMI classifications and family history of cancer. The CIR calculation formula is as follows:

$$ {\text{CIR}} = \frac{{\text{the number of breast cancer cases }}}{{\text{the number of observed person years}}} $$

$$ {\text{Upper Limit}} = {\text{CIR}} \times {\text{exp}}^{{1.96 \times \sqrt {1 \div {\text{actual number of breast cancer cases}}} }} $$

$$ {\text{Lower Limit}} = {\text{CIR}} \div {\text{exp}}^{{1.96 \times \sqrt {1 \div {\text{actual number of breast cancer cases}}} }} $$

After adjusting for covariates, Cox proportional hazard models were used to estimate the association of BMI and the joint effect between BMI and family history of cancer on breast cancer risk. The proportional hazard assumption for BMI and family history of cancer were evaluated using log–log survival plots^20,21^. The potential curvilinear relationship of BMI with breast cancer risk was assessed by restricted cubic splines (RCS) using the 5th, 50th, and 95th percentiles as fixed knots. The BMI equal to 24 kg/m^2^ was chosen as the reference group. A *p* value for nonlinearity < 0.05 suggested a nonlinear association between BMI and breast cancer.

The interactions between BMI and family history of cancer on breast cancer were measured using additive and multiplicative scales. The coefficient of the product term (BMI* family history of cancer) in the Cox regression model assesses the multiplicative interaction. Three indicators of additive interaction were used to evaluate the interaction between BMI and a family history of cancer: (1) the relative excess risk due to interaction (RERI), (2) the attributable proportion due to interaction (AP), and (3) the synergy index (SI), defined as follows^22^:

$$ RERI = HR_{11} - HR_{10} - HR_{01} + 1 $$

$$ AP = \frac{RERI}{{HR_{11} }} $$

$$ SI = \frac{{HR_{11} - 1}}{{\left( {HR_{10} - 1} \right) + \left( {HR_{01} - 1} \right)}} $$

Here HR_11_ represented the hazard ratio of women with BMI ≥ 24 and with family history of cancer, HR_10_ for women with BMI ≥ 24 and no family history of cancer, and HR_01_ for women with BMI < 24 and with family history of cancer. There was an additive interaction if RERI and AP were equal to 0 or S was unequal to 1. SAS version 9.4 software (SAS Institute, Cary, NC, USA) was used to analyze the data. All statistical analyses were two-sided, and a result for which *P* < 0.05 was considered statistically significant.

### Ethics approval and consent to participate

The study was approved by the Institutional Ethical Approval Committee of the Center for Disease Prevention and Control of Minhang district, Shanghai, China. The Institutional Review Board of the Center for Disease Prevention and Control in Minhang District, Shanghai, waived the requirement for informed consent from participants involved in the study, as the data analyzed in the study were compiled from anonymized data from electronic medical records. All methods were carried out by the relevant guidelines and regulations.

## Results

### Basic demographic characteristics

A total of 15,055 women were included in the analyses. Table 1 shows the demographic characteristics and lifestyle factors by baseline BMI categories. Among all participants, the median age was 55.87 years, and 34.0% were over 60 years old. Significant differences were found for age(*P* < 0.001), marriage status(*P* < 0.001), educational levels(*P* < 0.001), 3-month income levels(*P* < 0.001), diabetes status(*P* < 0.001), frequency of eating preserved food per week(*P* = 0.025) and frequency of eating high-fat food per week(P < 0.001) across BMI categories.

**Table 1:** Baseline characteristics and lifestyle variable in subjects.

Demographic and clinical characteristics	Total (N = 15,055)	BMI (China category, kg/m^2^)^a^				*P* values^b^
		Underweight (N = 697)	Normal weight (N = 5420)	Overweight (N = 6255)	Obesity (N = 2683)	
Age of screening						
Median, IQR	55.87 (48.38,62.31)	53.45 (41.39,61.26)	54.09 (44.91,60.98)	56.42 (49.96,62.37)	58.62 (52.28,64.60)	< 0.001
Age groups, N (%)						
< 60	9936 (66.00)	506 (72.60)	3863 (71.27)	4057 (64.86)	1510 (56.28)	< 0.001
≥ 60	5119 (34.00)	191 (27.4)	1557 (28.73)	2198 (35.14)	1173 (43.72)	
Marriage status, N (%)						
Married/remarried	13,954 (92.69)	640 (91.82)	5079 (93.71)	5819 (93.03)	2416 (90.05)	< 0.001
Divorced/widowed/unmarried	1101 (7.31)	57 (8.18)	341 (6.29)	436 (6.97)	267 (9.95)	
Educational levels, N (%)						
Primary	3516 (23.35)	88 (12.63)	866 (15.98)	1571 (25.12)	991 (36.94)	< 0.001
Secondary	9684 (64.32)	468 (67.14)	3659 (67.51)	4025 (64.35)	1532 (57.10)	
Tertiary	1855 (12.32)	141 (20.23)	895 (16.51)	659 (10.54)	160 (5.96)	
Three-month income levels, N (%)						
< 2000¥	4392 (29.17)	159 (22.81)	1449 (26.73)	1852 (29.61)	932 (34.74)	< 0.001
2000–4000¥	6487 (43.09)	278 (39.89)	2340 (43.17)	2721 (43.50)	1148 (42.79)	
≥ 4,000¥	4176 (27.74)	260 (37.3)	1631 (30.09)	1682 (26.89)	603 (22.47)	
Diabetes, N(%)						
Yes	1525 (10.13)	32 (4.59)	364 (6.72)	690 (11.03)	439 (16.36)	< 0.001
No	13,530 (89.87)	665 (95.41)	5056 (93.28)	5565 (88.97)	2244 (83.64)	
Family history of cancer						
Yes	4210 (27.96)	191 (27.40)	1479 (27.29)	1798 (28.75)	742 (27.66)	0.339
No	10,845 (72.04)	506 (72.60)	3941 (72.71)	4457 (71.25)	1941 (72.34)	
Smoking, N (%)						
Yes	160 (1.06)	13 (1.87)	43 (0.79)	75 (1.20)	29 (1.08)	0.184
No	14,895 (98.94)	684 (98.13)	5377 (99.21)	6180 (98.99)	2654 (98.92)	
Drinking, N (%)						
Yes	478(3.18)	20(2.87)	180(3.32)	210(3.36)	68(2.53)	0.165
No	14,577(96.82)	677(97.13)	5240(96.68)	6045(96.64)	2615(97.47)	
Daily Fruit and vegetable intake, N (%)						
< 300 g	2408 (15.99)	111 (15.93)	892 (16.46)	952 (15.22)	453 (16.88)	0.155
≥ 300 g	12,647 (84.01)	586 (84.07)	4528 (83.54)	5303 (84.78)	2230 (83.12)	
Frequency of eating preserved food per week, N (%)						
At least once	5186 (34.45)	205 (29.41)	1895 (34.96)	2141 (34.23)	945 (35.22)	0.025
Not even once	9869 (65.55)	492 (70.59)	3525 (65.04)	4114 (65.77)	1738 (64.78)	
Frequency of eating fried and smoked food per week, N (%)						
At least once	3465 (23.02)	162 (23.24)	1251 (23.08)	1457 (23.29)	595 (22.18)	0.712
Not even once	11,590 (76.98)	535 (76.76)	4169 (76.92)	4798 (76.71)	2088 (77.82)	
Frequency of eating high-fat food per week, N (%)						
At least once	6564 (43.60)	261 (37.45)	2189 (40.39)	2847 (45.52)	1267 (47.22)	< 0.001
Not even once	8491 (56.40)	436 (62.55)	3231 (59.61)	3408 (54.48)	1416 (52.78)	

^a^China category of BMI: underweight, < 18.5 kg/m^2^; normal weight, 18.5–23.9 kg/m^2^; overweight, 24–27.9 kg/m^2^; and obesity, ≥ 28 kg/m^2^.

^b^*P* < 0.05 was considered statistically significant.

### Breast cancer incidence and hazard ratio by BMI and family history of cancer

**Figure 1:**
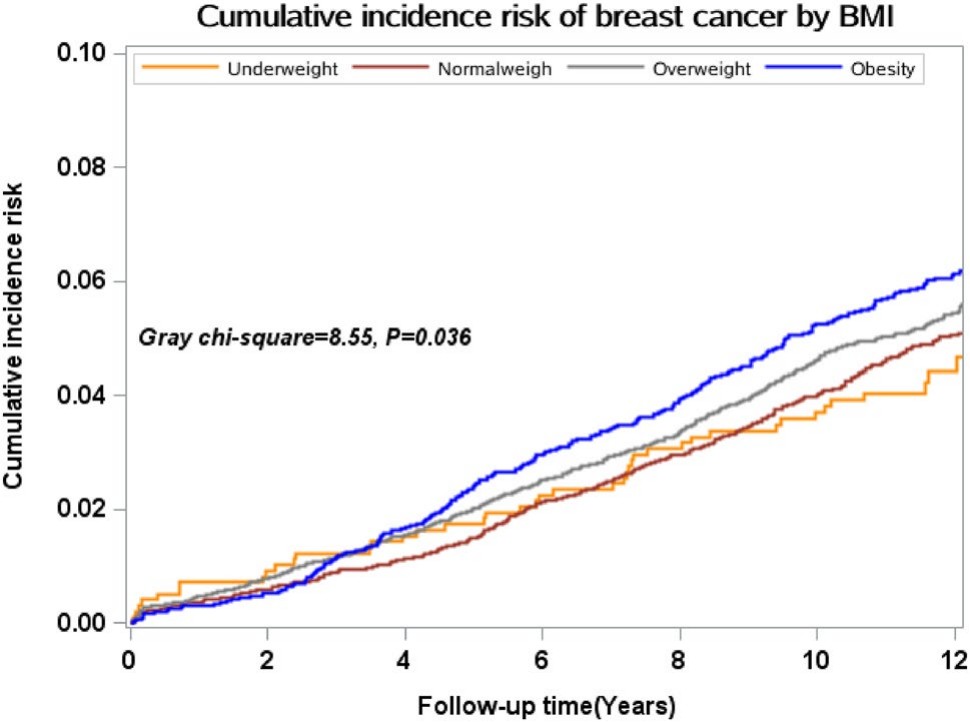
Cumulative incidence risk of breast cancer according to BMI classification in China.

As shown in Table 2, after 174,281.91 person-years of follow-up, 196 participants were diagnosed with breast cancer, giving rise to a CIR of 110.74 (95% CI 96.19, 127.52)/100,000 person-years. The incidence risk of breast cancer in the obese group was highest, with a CIR of 166.32 (95% CI 126.73, 218.26)/100,000 person-years, which was 2.09 (95% CI 1.42, 3.07) times the incidence risk of the normal weight group after adjusting for other variables. The breast cancer incidence risk of participants with cancer family history was 1.63 (95% CI 1.22, 2.49) times the incidence risk of participants without family history of cancer after adjusting for other variables, with CIR of 163.08 (95% CI 130.81, 203.32)/100,000 person-years. Figure 1 shows cumulative incidence risk of breast cancer estimates based on baseline BMI categories after accounting for competing risks (Gray chi-square = 8.55, *P* = 0.036).

**Table 2:** Breast cancer incidence by baseline BMI and family history of cancer.

Factors	No. of subjects	No. of cases	Person-years	CIR (95%CI) (1/100,000 person-years)	HR (95% CI)^1^
BMI (China category, kg/m^2^)					
< 18.5	697	8	8026.07	99.68 (49.85, 199.31)	1.05 (0.50, 2.21)
18.5–23.9	5420	58	62,517.10	92.77 (71.72, 120.06)	1 (reference)
24–27.9	6255	78	72,472.83	107.63 (86.20, 134.37)	1.24 (0.88, 1.75)
≥ 28	2683	52	31,265.91	166.32 (126.73, 218.26)	2.09 (1.42, 3.07)
Family history of cancer					
Yes	4210	79	48,441.97	163.08 (130.81, 203.32)	1.63 (1.22, 2.49)
No	10,845	117	125,839.90	92.98 (77.57, 111.45)	1 (reference)
Overall	15,055	196	174,281.91	110.74 (96.19, 127.52)	

*HR:Hazard ratio adjusted for age(< 60, ≥ 60);marriage status(in marriage ,not in marriage);education(primary, secondary, tertiary); personal monthly income (< 2000¥, 2000–4000¥, > 4000¥);smoking (yes, no);drinking (yes, no);daily fruit and vegetable intake (< 300, > 300 g/day); frequency of preserved food per week(at least once, not even once); frequency of fried and smoked food per week(at least once, not even once); frequency of high-fat food per week(at least once, not even once);family history of cancer (yes, no);diabetes(yes, no).

**Figure 2:**
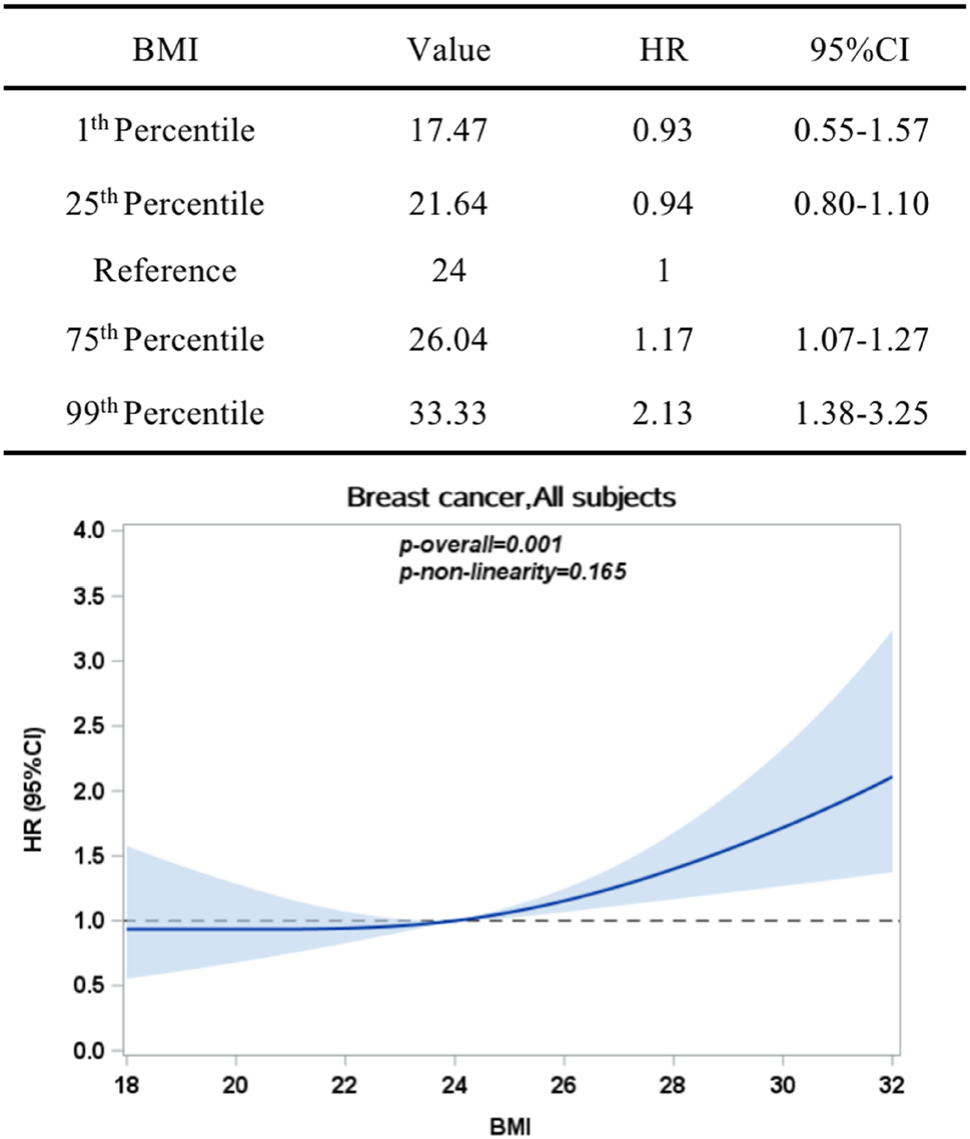
HR (95% CIs) between BMI (kg/m^2^) and breast cancer, allowing non-linear effects (adjusted for age; marriage status; education; personal monthly income; smoking; drinking; daily fruit and vegetable intake; frequency of eating preserved food per week; frequency of eating fried and smoked food per week; frequency of eating high-fat food per week; diabetes; family history of cancer). The reference BMI (with HR fixed as 1.0) was 24 kg/m^2^.

**Figure 3:**
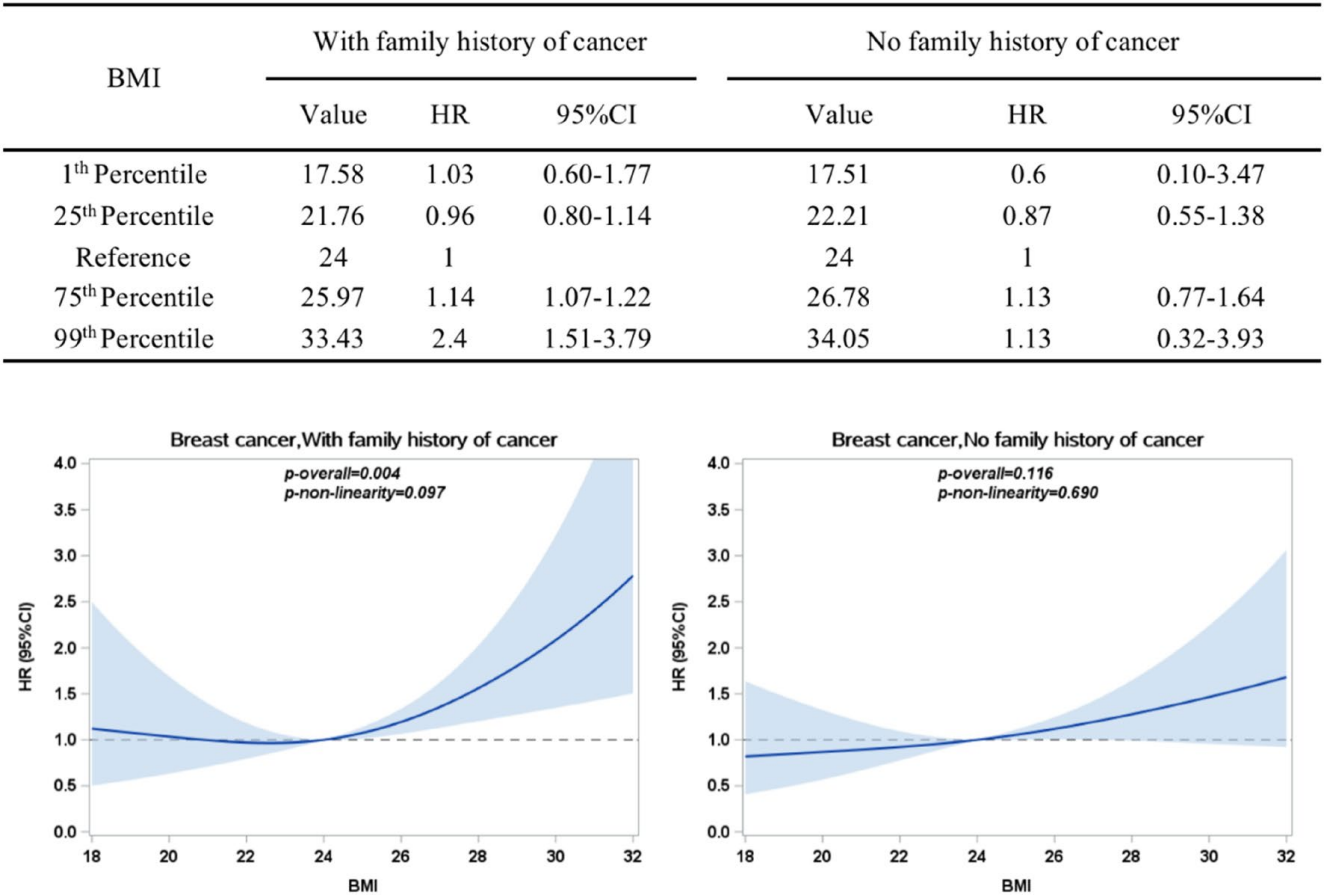
HR (95% CIs) between BMI (kg/m^2^) and breast cancer, stratified by family history of cancer, allowing non-linear effects (adjusted for age; marriage status; education; personal monthly income; smoking; drinking;daily fruit and vegetable intake; frequency of eating preserved food per week; frequency of eating fried and smoked food per week; frequency of eating high-fat food per week; diabetes). The reference BMI (with HR fixed as 1.0) was 24 kg/m^2^.

As shown in Fig. 2, BMI was positively associated with breast cancer (*P*_*overall*_ = 0.001) in a linear pattern (*P*_*nonlinearity*_ = 0.165). Statistically speaking, there was no correlation between BMI and breast cancer when BMI was less than 24 kg/m^2^. However, when BMI was greater than 24 kg/m^2^, breast cancer incidence risk rose as BMI increased. As shown in Fig. 3, BMI was significantly related to the risk of breast cancer for women with a family history of cancer (*P*_*overall*_ = 0.004) in a linear pattern (*P*_*nonlinearity*_ = 0.097). Similarly, after BMI was greater than 24 kg/m^2^, BMI was positively correlated with the incidence of breast cancer. However, for women without a family history of cancer, BMI was not statistically associated with breast cancer risk (*P*_*overall*_ = 0.116).

### Interaction and Joint effect between BMI and family history of cancer

Table 3 shows the joint association between BMI and family history of cancer. The statistical association between family history of cancer and breast cancer incidence only existed among women with BMI ≥ 24, but not among women with BMI < 24.Compared with BMI of < 24 kg/m^2^ and no family history of cancer, women with BMI of ≥ 24 kg/m^2^ and a family history of cancer had a higher risk of breast cancer, with a CIR of 186.54 (95% CI 138.25, 251.53) and the adjusted hazards ratio was 2.06 (95% CI 1.39, 3.06). We found a positive additive interaction between BMI and family history of cancer, with the RERI being 0.29 (95% CI 0.08, 0.51) and the AP being 0.37 (95% CI 0.08, 0.66). The multiplicative interactions were not statistically significant (HR:1.25; 95% CI 0.71, 1.65).

**Table 3:** Joint association of BMI and family history of cancer on the incidence of breast cancer.

Joint effect		No. of cases	CIR (95%CI) (1/100,000 person-years)	HR (95% CI) *	*P* value
Joint effect of different exposure combinations					
BMI < 24 (Underweight and normal weight)	No family history of cancer	61	92.36 (71.86, 118.71)	1 (reference)	
	With family history of cancer	36	141.78 (102.27, 196.56)	1.48 (0.98, 2.34)	0.062
BMI ≥ 24 (Overweight and obesity)	No family history of cancer	56	92.65 (72.07, 121.69)	1.12 (0.78, 1.62)	0.540
	With family history of cancer	43	186.54 (138.25, 251.53)	2.06 (1.39, 3.06)	0.000
Interaction on multiplicative scale				Estimate (95%CI)	*P* value
BMI*Family history of cancer				1.25 (0.71, 1.65)	0.443
Interaction on additive scale				Estimate (95% CI)	*P* value
RERI				0.29 (0.08, 0.51)	0.007
AP				0.37 (0.08, 0.66)	0.047
SI				0.41 (0.06, 2.83)	0.766

*Adjusted for age (< 60; ≥ 60 years), education (elementary, secondary, tertiary), personal monthly income (< 2000¥, 2000–4000¥, > 4000¥), marriage status(in marriage, not in marriage), smoking (yes, no), drinking (yes, no), daily fruit and vegetable intake (< 300, > 300 g/day), frequency of preserved food per week(at least once, not even once); frequency of fried and smoked food per week(at least once, not even once); frequency of high-fat food per week(at least once, not even once);family history of cancer (yes, no); BMI(< 24 kg/m^2^, ≥ 24 kg/m^2^).

## Discussion

In this population-based cohort study, we found that obesity and cancer family history were associated with a higher risk of breast cancer in females. Among 15,055 participants, when BMI ≥ 24, the risk of breast cancer increased as BMI increased. The same trend can be observed in women with family history of cancer, but not in women without family history of cancer, indicating that the association between BMI and breast cancer is affected by whether there is family history of cancer. Moreover, there was an interaction between overweight/obesity and cancer family history. When both risk factors were presented, the risk of breast cancer incidence was 2.06-fold that of neither risk factor (95% CI 1.39, 3.06). Although multiplicative interaction was not significant, a positive additive interaction between BMI and family history of cancer on breast cancer incidence was observed [RERI:0.29 (HR 0.08–0.51)], implying that BMI ≥ 24 and family history of cancer together may have amplified association on the incidence breast cancer compared to the sum of their individual associations.

The results of our study show that women who are overweight or obese have a significantly increased risk of breast cancer, which is consistent with the findings of other studies^7,23,24^.In postmenopausal women, a positive association between BMI and breast cancer risk has been observed in several studies^25,26^. Our findings are consistent with previous research results. Some studies have also shown that the incidence of breast cancer in women with family history is much higher than that in women without family history^27,28^, and family history of cancer other than family history of breast cancer is also related to the incidence of breast cancer^29^. However, there has been no report about the interaction and joint effect between BMI and cancer family on the risk of breast cancer in previous studies.

Our study revealed the additive interaction between BMI and cancer family history on the incidence of breast cancer. Although multiplicative interaction was not significant, we found a positive association between BMI and breast cancer incidence among women with a family history of cancer. The association between BMI and breast cancer incidence may be modified by a family history of cancer. Additive interaction measures the absolute change of risk, while multiplicative interaction measures the relative risk change. Additive interaction has more public health significance and is more related to biological interaction^30,31^

Based on the findings of our study, more efforts should be invested in promoting keeping fit as a way to reduce the risk of breast cancer among women with cancer family history. Therefore, reasonable weight control can not only reduce the risk of overweight and obesity on breast cancer but can also reduce the combined risk of BMI and cancer family history on breast cancer.

The mechanisms and causal pathways of obesity affecting the onset of breast cancer are complex. For example, increased estrogens, insulin resistance, mammary fat inflammation, increased aromatase expression, and elevated leptin levels are all thought to play a role in the pathogenesis of obesity-associated breast cancer^32–37^. The specific mechanism of the interaction between BMI and a family history of cancer has not yet been reported. However, there was evidence of an interaction between breast density and genetic risk measures, with low breast density more likely to carry high-risk breast cancer susceptibility mutations and an inverse association between breast density and BMI^38,39^. It may be one of the mechanisms by which BMI interacts with a family history of cancer in breast cancer incidence.

Overweight and obesity are modifiable risk factors, while family history of cancer is not. Nevertheless, the prevalence of obesity is on the rise in China as a result of the development of Chinese regional society and a change in people's lifestyles^40^. China has thus far been the country with the highest number of obese people, resulting in enormous health, economic, and social risks, making obesity prevention and control a very serious public health problem^41,42^. Overweight and obesity, in addition to being associated with breast cancer on their own, also interacted with a family history of cancer to increase breast cancer risk, according to our study. Therefore, interventions and treatments for overweight and obesity in women with a family history of cancer have high public health benefits.

This study analyzed the association between BMI and female breast cancer and combined it with restricted cubic splines to assess the potential nonlinear association between the two. Further analysis of the interaction between BMI and cancer family history on breast cancer risk provided new insights into the causal pathways of breast cancer incidence. Several limitations should also be noted. First, cancer family history was collected through face-to-face interviews, which may have induced some self-report and recall biases. Second, the variables of height and weight were only measured once at baseline, but the human body weight may change over the follow-up duration. In future studies, the variables should be measured multiple times to determine the relationship between body changes and breast cancer risk in women. Third, information about the use of hormones, menopausal age, and physical exercise of the women that may affect the conclusions of this study was not collected. In follow-up studies, we need to collect for further research.

## Conclusions

Our study suggested that obesity and family history of cancer together may have an amplified association on the risk of breast cancer compared to their independent association, highlighting the importance of keeping fit in women with family history of cancer, which suggested that obesity status should be given greater attention in high-risk groups with a family history of cancer and that obesity interventions in women with a family history of cancer may be able to provide additional health care benefits.

## Data Availability

The datasets generated and analyzed in the study are not publicly available but are available from the corresponding authors at reasonable request.
